# The nucleus tractus solitarii across vertebrates: developmental origins, comparative organization, and supranuclear modulation in humans

**DOI:** 10.3389/fnint.2026.1768344

**Published:** 2026-03-17

**Authors:** Yong-Shin Hong, Young-seok Park

**Affiliations:** Department of Oral and Maxillofacial Anatomy, Seoul National University, Seoul, Republic of Korea

**Keywords:** airway protection, brainstem, comparative neuroanatomy, interoception, neuromodulation, nucleus tractus solitarii, supranuclear modulation, swallowing

## Abstract

The nucleus tractus solitarii (NTS) is a highly conserved brainstem structure that has served as a principal hub for visceral sensory integration across vertebrate evolution. While the NTS has long been described as a relay for cardiovascular and respiratory reflexes, recent work increasingly frames it as an integrative node that transforms diverse afferent signals into adaptive, context-sensitive responses. In this review, we synthesize evidence on the developmental origins of the NTS (including contributions from the dorsal alar plate and epibranchial placodes) and its comparative organization across vertebrate taxa. We argue that many interspecies differences are more plausibly interpreted as functional reweighting within a conserved circuit framework—shaped by species-specific respiratory–feeding strategies and ecological demands—rather than as wholesale rewiring of the core network. Within this comparative context, the extensive supranuclear modulation observed in humans is discussed not as biological “superiority,” but as layered control that has become particularly prominent in response to human-specific anatomical constraints and behavioral demands, including those associated with speech and complex social interaction. Clinically, we revisit dysphagia, cough hypersensitivity, and nausea/vomiting as manifestations of network-level dysregulation and gating failure rather than isolated breakdowns of single reflex arcs. Finally, we suggest that neuromodulation strategies, including vagus nerve stimulation, may be best conceptualized as delivering patterned afferent input capable of shaping NTS network plasticity, rather than as non-specific electrical activation.

## Introduction

The nucleus tractus solitarii (NTS) is a major brainstem structure in which visceral and gustatory afferent information converges, primarily via cranial nerves VII, IX, and X (Andresen and Kunze, 1994; Saper, 2002). Traditionally, the NTS has been described in relation to its role in cardiovascular reflexes, respiratory control, swallowing, and airway protection (Dampney, 2016; Jean, 2001). These physiological functions are well established and remain fundamental to our understanding of brainstem organization.

At the same time, accumulating anatomical and functional evidence suggests that the NTS participates in a broader range of integrative processes than reflex transmission alone. Anatomically, it maintains strategic connections with branchial motor networks, the medullary reticular formation, and supranuclear systems involved in autonomic regulation and interoceptive processing (Craig, 2002; Saper, 2002). In humans, behaviors such as swallowing, phonation, airway regulation, gustation, and affect-related responses depend on this conserved brainstem sensory platform, while also being influenced by higher cortical and limbic modulation (Rinaman, 2011; Simonyan and Horwitz, 2011). Understanding how these levels interact remains an important question in systems neuroscience.

In this review, we revisit the NTS from an integrated perspective that brings together developmental lineage, comparative organization across vertebrates, and supranuclear modulation in humans. Rather than proposing a replacement for established physiological models, we aim to situate them within a broader framework that considers evolutionary conservation alongside species-specific adaptations. Within this context, differences observed across taxa are approached as variations in functional weighting within a preserved circuit framework.

**FIGURE 1:**
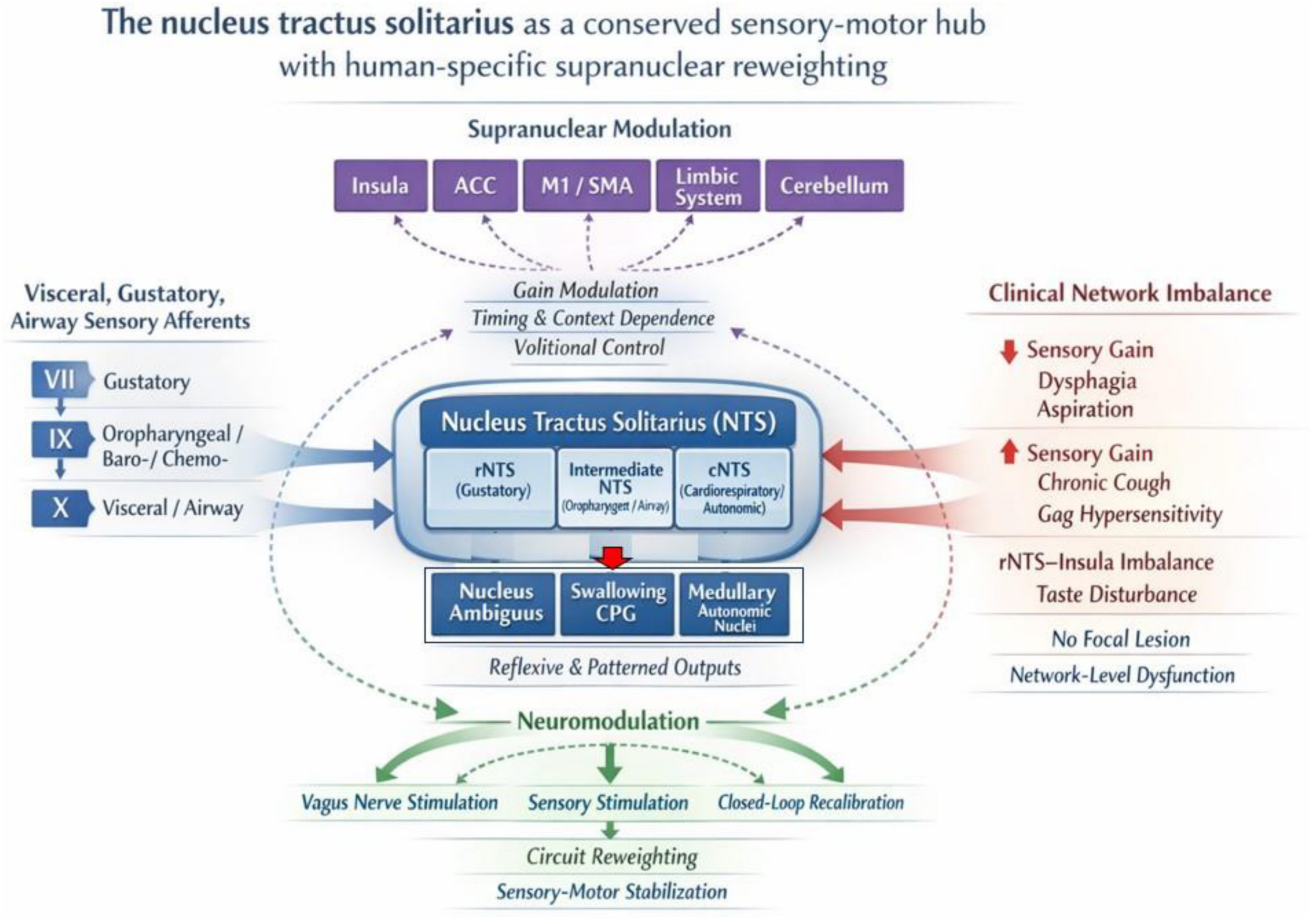
The nucleus tractus solitarii (NTS) as a Conserved Sensory-Motor Hub Subject to Supranuclear Reweighting. This schematic illustrates interactions between reflexive brainstem circuits and descending supranuclear influences. (Left) Heterogeneous sensory afferents (gustatory, oropharyngeal, visceral) from cranial nerves VII, IX, and X converge onto the viscerotopically organized NTS. (Top) Crucially, the NTS is not a passive relay but is subject to “Gain Modulation” by supranuclear structures (Insula, ACC, M1/SMA), allowing context-dependent and volitional control over reflexive outputs. (Right) Clinical pathologies are conceptualized as “Network Imbalances” of this sensory gain: downregulated gain leads to deficits such as dysphagia and aspiration, whereas upregulated gain results in hypersensitivity disorders like chronic cough. (Bottom) Therapeutic neuromodulation aims to reweight this circuit, stabilizing the sensory-motor integration through targeted sensory stimulation or vagus nerve stimulation.

We further consider clinical phenomena—including dysphagia, impaired airway protection, gag reflex abnormalities, and chronic cough—not solely as isolated failures of individual reflex arcs, but as potential expressions of imbalance within distributed NTS-centered networks. Finally, we briefly discuss how such a framework may inform contemporary approaches to neuromodulation, including vagus nerve stimulation (Groves and Brown, 2005), by conceptualizing them as patterned afferent inputs capable of influencing NTS network dynamics. The overall conceptual structure of this review is summarized in Figure 1.

## Developmental origins of the nucleus tractus solitarius

The nucleus tractus solitarii (NTS) originates from the dorsal alar plate of the embryonic brainstem, forming the core of the visceral sensory column. This developmental position is fundamentally distinct from that of the branchial motor nuclei—which give rise to the trigeminal, facial, and ambiguus nuclei—derived from the basal plate (Butler and Hodos, 2005; Nieuwenhuys et al., 2008). While classical neuroanatomy proposed the concept of “longitudinal columns” to suggest that sensory and motor systems develop in separate compartments from the outset, recent molecular genetic studies have demonstrated that these macroscopic divisions are established through precise transcriptional regulatory programs.

Experimental evidence indicates that the differentiation of branchial and visceral motor neurons is critically dependent on the homeobox gene *Phox2b* (Pattyn et al., 2000; Pla et al., 2008). In contrast, the fate of visceral sensory neurons comprising the NTS is demarcated and determined independently through interactions with transcription factors such as *Nkx2.2* and *Nkx2.9* (Chandrasekhar, 2004; Jarrar et al., 2015). Thus, although the NTS and branchial motor nuclei lie in close anatomical proximity within the adult brainstem, they follow genetically strictly separated lineages. While the NTS itself belongs to the sensory column, this molecular compartmentalization of the branchio-visceral axis provides the developmental background that allows the NTS to subsequently form precise connections with motor networks. In other words, sensory and motor components depart from developmentally separate origins but are molecularly primed to be functionally coupled through complementary programs (Table 1).

**TABLE 1:** Developmental origins and molecular determinants of the nucleus tractus solitarii (NTS).

Feature	Nucleus tractus solitarii (NTS)	Branchial motor nuclei
Embryonic origin	Dorsal alar plate	Ventral basal plate
Functional column	Visceral sensory column	Branchio-visceral motor column
Key transcription factors	*Nkx2.2*, *Nkx2.9*, *Tlx3* (sensory fate determination)	*Phox2b*, *Isl1*, *Tbx20* (motor fate determination)
Segmental organization	Rhombomeres r7–r11 (caudal hindbrain)	r2–r7 (migration to facial, trigeminal, and ambiguus nuclei)
Afferent input origin	Epibranchial placodes	(Not applicable—efferent output structures)
Adult connectivity	“Input Node” for sensory–motor integration	“Output Node” receiving processed input from the NTS

This table contrasts the developmental characteristics of the NTS with adjacent motor nuclei. The formation of the NTS is regulated by interactions between *Phox2b*, *Nkx2.2*, and *Nkx2.9*, establishing its identity as a visceral sensory column independent of the motor system (Butler and Hodos, 2005; Chandrasekhar, 2004; Jarrar et al., 2015; Nieuwenhuys et al., 2008; Pattyn et al., 2000; Pla et al., 2008; Thompson et al., 2014; Watson et al., 2019).

This molecular distinction has been further concretized anatomically through recent rhombomere-based mapping. Watson et al. (2019) proposed a modern nomenclature, identifying that the human NTS derives not from a vague dorsal medullary region, but from specific alar plate domains extending from rhombomere 7 to 11 (r7–r11) (Watson et al., 2019). Furthermore, developmental gene expression data from the Allen Brain Atlas suggest that neuronal identity within these segments is “pre-patterned” by conserved transcription factors (Thompson et al., 2014). This implies that the viscerotopy and circuit assembly of the NTS are not random processes, but results genetically programmed from the rhombomeric stage.

The origins of peripheral inputs to the NTS also reflect the characteristics of this sensory–motor dual lineage. Neurons of the geniculate, petrosal, and nodose ganglia, which convey gustatory and visceral information, originate from epibranchial placodes rather than the neural crest, extending their axons centrally into the NTS (Blentic et al., 2011; Schlosser, 2006; Streit, 2004). Developmentally, the NTS possesses an origin and input pathway independent of the branchial motor system, yet during maturation, it forms intimate synaptic connections with motor nuclei according to functional demands. This principle of developmental separation–functional integration implies that complex behaviors such as swallowing and airway protection are not simple reflexes, but sophisticated recombinations of neural networks with distinct origins (Jean, 2001).

**FIGURE 2:**
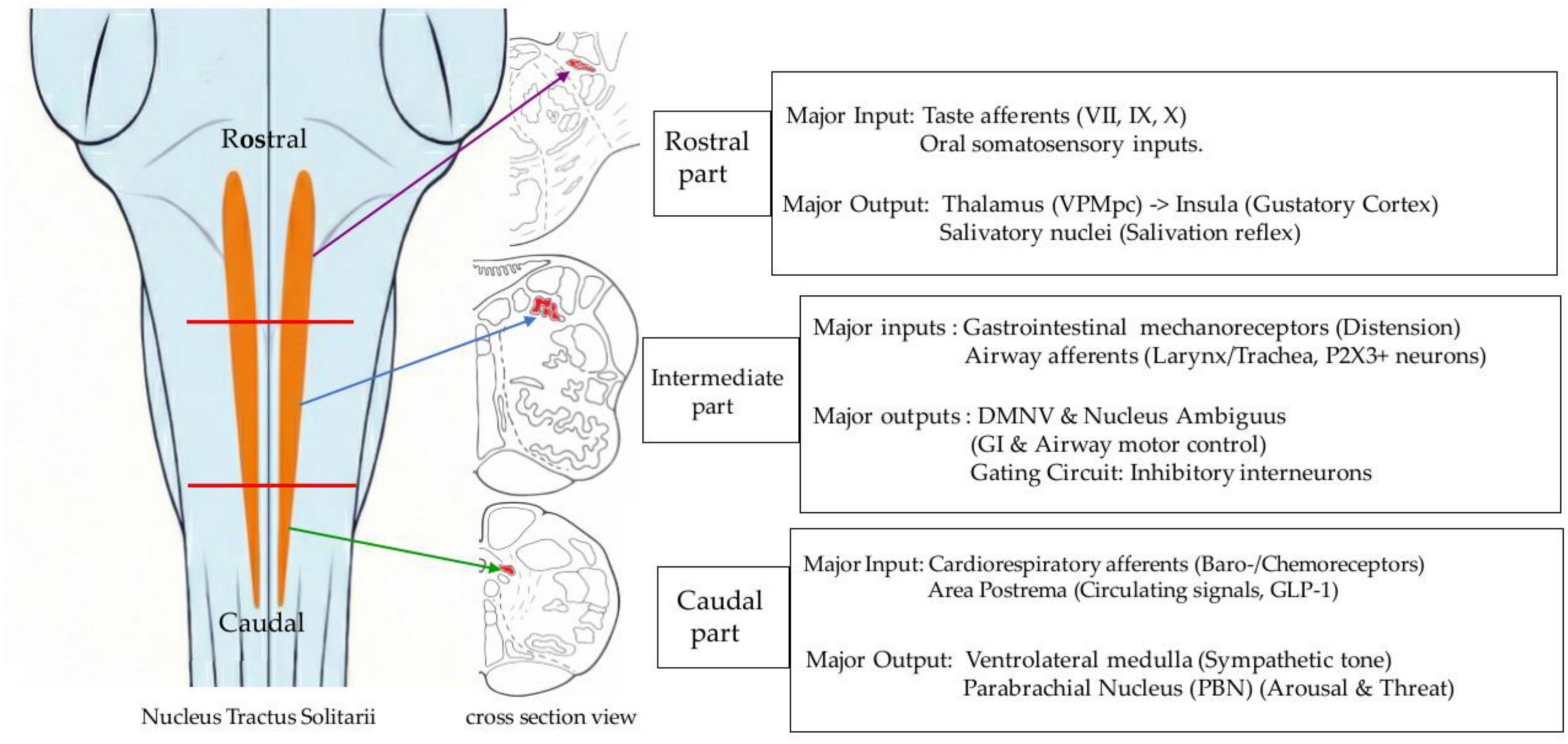
Rostrocaudal organization of the nucleus tractus solitarii and its principal input–output tendencies. This schematic illustrates the rostrocaudal organization of the nucleus tractus solitarii (NTS). The left panel depicts the longitudinal position of the NTS within the brainstem, while the right panel summarizes representative cross-sectional levels and the principal input–output tendencies associated with each subdivision. The rostral portion primarily receives gustatory afferents conveyed via the facial (VII), glossopharyngeal (IX), and vagus (X) nerves, as well as oral somatosensory inputs. Its ascending projections include pathways to the thalamus (ventral posteromedial nucleus, parvicellular part) and subsequently to the insular cortex, consistent with the gustatory pathway. Connections with salivatory nuclei are also implicated in salivary reflex regulation. The intermediate portion integrates laryngeal and pharyngeal afferents, including airway-related mechanosensory inputs. Projections to the nucleus ambiguus and the dorsal motor nucleus of the vagus contribute to the coordination of swallowing and airway protective reflexes. Local circuit mechanisms, including inhibitory interneurons, have been described in this region and may participate in modulating afferent transmission. The caudal portion receives cardiorespiratory afferents, including baroreceptor and chemoreceptor inputs, as well as visceral vagal signals from the gastrointestinal tract. It projects to the ventrolateral medulla and the parabrachial nucleus, structures involved in autonomic regulation and arousal-related processing. Functional interactions with the area postrema, which monitors circulating signals, have also been reported. It should be noted that this schematic is intended to summarize general functional tendencies. Recent viral tracing and single-cell studies indicate that afferent projections show substantial overlap and convergence rather than strict organ-based segregation. Accordingly, the rostrocaudal divisions depicted here represent relative functional distributions rather than sharply demarcated anatomical boundaries.

The internal organization of the adult NTS assembled through these developmental processes has traditionally been described as a viscerotopic structure arranged according to organ-specific afferents. However, recent anatomical and electrophysiological studies are refining this concept with greater precision. Organ-specific viral tracing by Bassi et al. (2022) revealed that vagal afferents from the gastrointestinal, cardiovascular, and respiratory systems are distributed broadly across multiple NTS subnuclei, rather than being confined to organ-exclusive subregions without overlap (Bassi et al., 2022). Moreover, Zhao et al. (2022) reported a multidimensional coding architecture in which single afferent neurons branch extensively within the NTS, and signals from different organs converge onto common second-order neurons (Zhao et al., 2022). This suggests that the NTS functions as an overlapping platform performing distributed integration from early stages, maintaining a topographical bias rather than a strict point-to-point relay. Consequently, these flexible structural features provide an ideal anatomical substrate capable of accommodating the functional conservation and species-specific differential emphasis discussed in the following section, rather than representing a rigidly fixed circuit. The rostrocaudal organization of the nucleus tractus solitarii and its principal input–output tendencies are schematically summarized in Figure 2.

## Comparative organization of the nucleus tractus solitarii across vertebrates

The multidimensional coding and distributed integration architecture of the nucleus tractus solitarii (NTS) described in the previous section illustrates how this nucleus has adapted to diverse environmental demands during evolution. While the fundamental structure and function of the NTS are highly conserved across vertebrate species, clear differences exist in the composition of sensory inputs and functional emphasis depending on the ecological and anatomical context of each species (Butler and Hodos, 2005). It is most plausible to interpret these variations not as a reconstruction of the fundamental circuit scaffold of the NTS itself, but as a result of relative weighting, where morphological variations in peripheral sensory organs and differences in respiratory and feeding strategies assign different weights to existing circuits (Table 2). In other words, variability from a comparative anatomical perspective reflects differences in which sensory modality is prioritized and integrated, rather than a difference in what the NTS does.

**TABLE 2:** Comparative anatomy and functional emphasis of the NTS across vertebrates.

Taxon	Key anatomical feature	Functional emphasis of NTS
Fish	Branchial pump; no distinct upper/lower airway	Aquatic respiration, gustatory–feeding reflexes, chemical sensory integration
Amphibians	Transition to air breathing; simple glottis	Integration of lung inflation signals; early coordination of buccal pumping and breathing
Reptiles	Fully established pulmonary respiration; airway protection structures	Differentiation of upper vs. lower airway inputs; enhanced separation of breathing and swallowing
Birds	Syrinx; air sac system	Precise control of phonation–respiration; conservation of topographic visceral maps
Mammals	Complex oropharyngeal structures; diaphragmatic breathing	Sophisticated suck–swallow–breathe coordination; high-level airway protection
Marine mammals	Blowhole; diving adaptations	Extreme reinforcement of cardiovascular–respiratory circuits for the diving reflex

This table summarizes how NTS circuits have been functionally reweighted according to species-specific survival strategies. While the fundamental circuit scaffold is conserved, the priority of sensory inputs has shifted to adapt to environmental demands (Arends et al., 1988; Bassi et al., 2022; Blentic et al., 2011; de Lahunta et al., 2015; Gargaglioni and Milsom, 2007; Katz and Karten, 1983; Kent and Carr, 2001; Nilsson, 2011; Schlosser, 2006; Taylor et al., 2010; Zhao et al., 2022).

In fish, the NTS is closely associated with the branchial pump, integrating chemical and mechanical external sensory signals related to feeding and respiration (Nilsson, 2011; Taylor et al., 2010). At this stage, the distinction between the upper and lower airways is not clearly defined, so the concept of airway protection specific to terrestrial vertebrates has not fully differentiated. However, the fundamental organizational principle—that sensory information converges along the solitary tract to interface with medullary autonomic and motor circuits—is already established (Kent and Carr, 2001). In amphibians, the transition to air breathing increases the importance of upper airway sensory inputs and pulmonary stretch signals. Consequently, the NTS undergoes adaptive expansion in a direction that integrates signals from the oral and pharyngeal regions to minimize conflict between swallowing and respiration (Gargaglioni and Milsom, 2007).

In the reptilian stage, as pulmonary respiration becomes fully established, the differentiation of processing for upper and lower airway sensory inputs becomes prominent, serving as the neurological substrate for airway protective behaviors (Taylor et al., 2010). Birds possess a unique vocal organ called the syrinx (instead of a larynx) and an air sac system, yet the NTS remains a key hub for life-sustaining functions. Notably, the classical study by Katz and Karten (1983) demonstrated the existence of a topographic visceral sensory map within the avian NTS (Katz and Karten, 1983). Furthermore, Arends et al. (1988) revealed that this circuit projects beyond the medullary reticular formation to broad higher centers, including the parabrachial complex, hypothalamus, and limbic forebrain (Arends et al., 1988). These sophisticated input–output structures suggest that, from early evolutionary stages, the NTS was not merely a passive relay for autonomic reflexes but an integrative hub for higher-order visceral information processing.

In mammals, branchial arch-derived structures such as the pharynx and larynx become highly complex, developing into behaviors where swallowing, breathing, and phonation are tightly integrated (de Lahunta et al., 2015; Haines and Mihailoff, 2018). The coordination of suck–swallow–breathe in early life exemplifies sensory–motor integration, providing the developmental basis for sophisticated oropharyngeal function in adulthood (Jean, 2001). Meanwhile, marine mammals exhibit unique respiratory adaptations, such as strategies to maintain cerebral perfusion and bradycardia during the diving reflex, or breathing through a blowhole. These represent excellent examples where the NTS–medullary–autonomic functional linkage has undergone extreme functional reweighting to suit specific peripheral sensory contexts (Panneton and Gan, 2020; Panneton et al., 2012; Panneton, 2013).

In summary, the NTS has consistently functioned across vertebrates as a central platform where sensory information converging along the solitary tract is integrated. These patterns of comparative anatomical conservation and variation provide an essential reference framework for interpreting the functional expansion (volitional control and supranuclear modulation) in humans, which will be discussed in the next section.

## Conserved functional roles of the nucleus tractus solitarii across vertebrates

Across vertebrate species, the nucleus tractus solitarii (NTS) consistently serves as a central node for sensory–motor integration essential for survival. This extensive conservation pattern implies more than the simple maintenance of anatomical structures; it signifies the preservation of a fundamental integrative principle that transforms sensory inputs into appropriate autonomic and motor outputs (Andresen and Kunze, 1994; Dampney et al., 2002; Zoccal et al., 2014). Although specific behavioral contexts vary among taxa, several key functional domains recur with notable regularity (Table 3).

**TABLE 3:** Conserved core physiological functions of the NTS across vertebrates.

Functional domain	Key sensory inputs	Conserved output and role
Interoception	Baroreceptors, chemoreceptors, pulmonary stretch	Maintenance of cardiorespiratory homeostasis
Swallowing	Mechanical/chemical stimuli from mouth, pharynx, larynx	Simultaneous execution of nutritive intake and airway protection (nutritive/protective synergy)
Airway defense	Noxious stimuli (water, acid, particles) in upper airway	Initiation of expulsive reflexes (laryngeal closure, cough, sneeze)
Energy balance	Gastric distension, glucose, GLP-1, cytokines	Regulation of feeding; Induction of adaptive sickness behavior

This table illustrates the fundamental integrative principles of the NTS that have remained consistent throughout evolution (Bailey et al., 2008; Barkmeier et al., 2000; Bieger, 1993; Bonham et al., 2006; Broussard and Altschuler, 2000; Cunningham and Sawchenko, 2000; Dampney et al., 2002; Grill and Hayes, 2012; Haines and Mihailoff, 2018; Holt, 2022; Jean et al., 1975; Northcutt, 2002; Panneton and Gan, 2020; Panneton et al., 2012; Panneton, 2013; Prescott and Liberles, 2022; Schwartz, 2000; Shao et al., 2024; Zoccal et al., 2014).

One of the most defining conserved functions is the integration of interoceptive signals. Critical physiological variables such as arterial pressure, blood gas levels, and pulmonary stretch converge upon the NTS, primarily via the glossopharyngeal (IX) and vagus (X) nerves (Andresen and Kunze, 1994; Bieger, 1993). While the location and sensitivity of peripheral receptors may differ across species, the central mechanism transmitting these signals to autonomic regulatory zones within the medulla to maintain physiological homeostasis remains highly stable (Jean et al., 1975).

Swallowing and airway protection constitute another core axis of this conserved circuitry. Swallowing is not merely the transport of a bolus but a complex reflex behavior requiring tight coordination with airway protection and respiration; it relies entirely on the precise entry of sensory signals from the oral cavity, pharynx, and upper airway into the NTS (Barkmeier et al., 2000; Broussard and Altschuler, 2000). Similarly, airway defensive responses such as laryngeal closure, coughing, and gagging are based on a shared circuit wherein upper airway stimuli are converted into motor outputs via the NTS (Bonham et al., 2006; Schwartz, 2000). Differences in response intensity or behavioral expression across species are not due to a lack of NTS circuitry, but rather the result of functional reweighting adapted to specific upper airway anatomy and behavioral contexts.

Furthermore, the NTS acts as an evolutionarily ancient gateway regulating energy and metabolic homeostasis. Recent evidence indicates that the NTS goes beyond passive monitoring of metabolic indices (e.g., glucose) to drive adaptive homeostasis. As emphasized by Holt (2022), GLP-1 (glucagon-like peptide-1)–producing neurons within the NTS detect interoceptive stress—such as systemic inflammation, hypoxia, and visceral pain—and relay these signals to the parabrachial nucleus and hypothalamus. This pathway induces sickness behavior, characterized by appetite suppression and autonomic readjustment during stress, representing a powerful conserved mechanism that allows the organism to defend physiological stability even amidst metabolic crises (Grill and Hayes, 2012; Holt, 2022; Northcutt, 2002).

Recent transcriptomic analyses and circuit-tracing studies have demonstrated that this functional conservation is organized at a highly sophisticated network level, beyond individual reflex arcs. Building on the developmental foundation (r7–r11 rhombomeric patterning) discussed in Section “Developmental origins of the nucleus tractus solitarius,” the NTS is composed of genetically defined cell types that are highly conserved across mammals, ensuring the stability of essential reflexes like airway protection and digestion (Prescott and Liberles, 2022; Watson et al., 2019). Moreover, the work of Cunningham and Sawchenko (2000), which meticulously traced the anatomical substrate of oromotor reflexes, highlights the complexity of NTS output networks. They revealed that NTS-mediated behaviors rely not on simple monosynaptic loops, but on polysynaptic pathways projecting to distributed premotor neuronal groups within the reticular formation, including the dorsal medullary reticular column (DMRC) (Cunningham and Sawchenko, 2000). These dorsal medullary pathways provide the substantial anatomical basis for a central pattern generator (CPG), where sensory input is spatiotemporally patterned before reaching motor pools of the jaw, tongue, pharynx, and larynx. In this framework, the NTS functions as a sensory integration and gating node positioned at the top of a distributed premotor network, sequentially recruiting multiple cranial nerve motor nuclei.

This gating function is exquisitely controlled by highly organized local circuits within the NTS, particularly GABAergic inhibitory interneurons. Bailey et al. (2008) confirmed that these inhibitory neurons are distributed across all NTS subnuclei and are synchronized with solitary tract input (Bailey et al., 2008), while Shao et al. (2024) demonstrated that they fine-tune respiratory and autonomic functions through extensive whole-brain input–output networks (Shao et al., 2024). Consequently, intrinsic inhibitory circuits within the NTS serve as gatekeepers, filtering out physiological noise and transmitting only salient visceral sensory signals to higher centers (Prescott and Liberles, 2022).

## Human-specific specialization of the NTS

While the NTS retains the conserved sensory–motor integration architecture found across vertebrates, in humans, this basic framework is subject to pronounced supranuclear modulation from cortical, limbic, and cerebellar systems. This expansion should not be interpreted as biological superiority over other mammals. Rather, it is best understood as a functional reconfiguration required to adapt to unique evolutionary and anatomical constraints, such as the descent of the larynx for speech and the demands of complex social interaction (Craig, 2009; Damasio and Carvalho, 2013; Saper, 2002; Table 4).

**TABLE 4:** Human-specific functional specialization and supranuclear modulation.

Specialization area	Evolutionary/anatomical driver	Expanded role of NTS
Volitional control	Strengthening of corticobulbar tracts	Conscious suppression/facilitation of reflexes; regulation of sensory gating
Speech and phonation	Laryngeal descent	Maintenance of respiratory rhythm during phonation; Fine coordination with vocal fold control
Flavor and emotion	Complex dietary culture and sociality	Transmission of gustatory signals to amygdala/insula to form emotional flavor experiences
Higher airway protection	Increased aspiration risk (crossed airway/foodway)	Proactive defense linked to cognitive sensing (e.g., urge-to-cough)

This table demonstrates the impact of human language and social behavior on NTS circuits. The key feature is context-dependent regulation extending beyond simple reflexes (Ackermann and Riecker, 2004; Canna et al., 2023; Canning et al., 2014; Craig, 2009; Damasio and Carvalho, 2013; Fitch, 2000; Hamdy et al., 1999; Humbert and Robbins, 2008; Levinson et al., 2023; Mazzone and Undem, 2016; Moe et al., 2024; Small and Prescott, 2005).

One of the most distinguishing features of this human-specific expansion is the increase in volitional modulation. Humans possess the ability to consciously initiate or suppress basic reflex behaviors, such as swallowing or coughing, according to contextual needs. This capacity stems from potent and continuous descending influences from higher centers, including the primary motor cortex (M1), supplementary motor area (SMA), and insula (Ackermann and Riecker, 2004; Hamdy et al., 1999; Simonyan and Horwitz, 2011). Such supranuclear control modulates the gain of sensory inputs, allowing the same peripheral stimulus to be flexibly translated into behavioral responses with variable timing and probability depending on the situation.

Phonation and speech production represent areas where this specialization is particularly evident. The descent of the larynx expanded the vocal resonance space but simultaneously created a precarious anatomical arrangement where respiratory and alimentary pathways intersect (Fitch, 2000). Consequently, during speech, sensory information generated from the mucosa of the larynx and upper airway engages the NTS, which must coordinate with the nucleus ambiguus and respiratory centers to support stable airway protection and respiratory rhythms even during continuous vocalization (Humbert and Robbins, 2008; Simonyan and Horwitz, 2011). This suggests that in humans, the NTS functions not merely as a trigger for reflexes, but as an active stabilizing element that maintains complex, ongoing sensory–motor sequences.

The processing of gustatory and oral sensory information also shows qualitative expansion in humans. Gustatory signals integrated within the rostral NTS (rNTS) ascend via pathways involving the thalamus, insula, and anterior cingulate cortex (ACC), where they are integrated into emotional and mnemonic experiences (flavor perception) beyond simple nutritive signaling (Craig, 2009; Small and Prescott, 2005). Recent high-resolution functional imaging and network analyses strongly support this view. Ultra-high-field (7T) fMRI studies have successfully visualized the role of the NTS as a primary gustatory relay in living humans (Canna et al., 2023), while structural connectivity analyses using Human Connectome Project data have revealed direct pathways linking the human NTS with limbic structures, including the amygdala and dorsolateral prefrontal cortex (DLPFC) (Levinson et al., 2023). These findings support the possibility that the human NTS possesses the anatomical substrate to integrate complex social and emotional cues directly into physiological responses.

In the context of airway protection, the human NTS exhibits highly sensitive regulatory capabilities. Canning et al. (2014) and Mazzone and Undem (2016) have redefined the NTS within a modern framework as a complex integrative hub with distinct inhibitory gating mechanisms. According to this theory, the NTS acts as a brainstem gate, actively filtering peripheral afferent inputs via synaptic inhibition and controlling the transmission of sensory signals to higher brain centers. Recent human functional imaging research (2024) has empirically demonstrated that failure of this gating mechanism at the medullary level is associated with cough hypersensitivity and pathological responses (Moe et al., 2024). Thus, human airway defensive responses should be viewed not as rigid reflexes, but as regulated defensive behaviors shaped by contextual and emotional factors from higher centers, with the NTS serving as the central node.

In summary, the functional characteristics of the human NTS are consistently explained not by the emergence of entirely new nuclei, but by the evolutionary expansion of supranuclear regulatory networks layered upon conserved brainstem circuits (Damasio and Carvalho, 2013; Saper, 2002). This perspective provides a theoretical foundation for interpreting clinical phenomena such as dysphagia, voice disorders, and cough hypersensitivity not as isolated failures of single reflexes, but as imbalances within an integrated supranuclear–brainstem control system.

## Clinical pathophysiology: network-level dysregulation centered on the nucleus tractus solitarii

The nucleus tractus solitarii (NTS) functions as the primary convergence site for peripheral sensory inputs and as a premotor initiation node intersecting with supranuclear regulatory networks. Consequently, dysfunction in this region tends to manifest not as isolated symptoms but as complex syndromes encompassing swallowing, airway protection, affect, and autonomic regulation (Benarroch, 1993; Hamdy et al., 1998; Jafari et al., 2003). Importantly, these clinical presentations do not necessarily stem solely from focal structural lesions within the brainstem (e.g., stroke or tumor). Rather, they are often better interpreted as network-level dysregulation resulting from diminished supranuclear control, damage or inflammation of peripheral sensory nerves, or failure to regulate sensory gain within the nucleus tractus solitarii circuitry (Table 5).

**TABLE 5:** Clinical manifestations of network-level dysregulation in NTS circuits.

Clinical syndrome	Pathophysiology	Network-level deficit
Dysphagia	Stroke, aging, neurodegenerative disease	Delayed sensory–motor integration; synchronization failure with swallowing CPG
Chronic cough	Post-viral, idiopathic	Loss of inhibitory gating within NTS; weakened cortical suppression
Nausea/emesis	Drug side effects, systemic illness	Excessive activation of area postrema and distributed emetic CPG
Silent aspiration	Parkinson’s disease, dementia	Failure to detect upper airway input; abnormally elevated reflex threshold
Dysgeusia	Peripheral nerve injury, infection	Maladaptive reconfiguration of rostral NTS and higher gustatory circuits

This table reinterprets clinical symptoms as network-level dysregulation rather than isolated structural failures (Benarroch, 1993; Doty, 2019; Hamdy et al., 1997, 1998; Humbert and German, 2013; Jafari et al., 2003; Langmore et al., 1998; Miller, 1982; Morice et al., 2014; Sanger and Andrews, 2006; Zhang et al., 2021).

One of the most frequently observed clinical manifestations is dysphagia and resultant aspiration. The normal initiation of swallowing predicates that sensory information from the oral cavity, pharynx, and upper airway reaches the nucleus tractus solitarii with precise timing and integration. Consequently, dysphagia observed during recovery from stroke, in neurodegenerative diseases, or associated with age-related cognitive decline is described less as simple weakness of motor muscles and more as a synchronization failure among the nucleus tractus solitarii, the central pattern generator for swallowing, and cortical control axes (Hamdy et al., 1997; Miller, 1982; Small and Prescott, 2005). This breakdown in sensorimotor coupling leads to delayed airway protective responses, sharply increasing the risk of aspiration (Langmore et al., 1998).

The spectrum of airway protective reflexes, including cough and the gag reflex, also provides critical insight into pathophysiology centered on the nucleus tractus solitarii. The cough reflex involves sequential activation of upper airway stimulation, the nucleus tractus solitarii, respiratory centers, and spinal motor pathways. In patients with chronic cough or cough hypersensitivity syndrome, the inhibitory gating function within brainstem circuits, including the nucleus tractus solitarii, is compromised. This results in coughing triggered by trivial stimuli (allotussivity) or at low thresholds (hypertussivity) (Canning et al., 2014; Mazzone and Undem, 2016; Morice et al., 2014). This reflects an excessive up-regulation of sensory gain within the nucleus tractus solitarii circuitry. Conversely, in degenerative conditions such as Parkinson’s disease, this gating threshold becomes abnormally elevated, leading to attenuation of the cough reflex and potentially fatal silent aspiration.

It is clinically crucial to distinguish the mechanisms of the gag reflex from those of nausea and vomiting. Unlike the gag reflex, in which mechanical stimulation of the pharyngeal mucosa triggers sequential muscle contraction via the nucleus tractus solitarii, nausea and vomiting are the domain of a distributed emetic central pattern generator that recruits respiratory and abdominal muscles in response to gastrointestinal toxins or systemic metabolic disturbances, as established by Sanger and Andrews (2006). Nausea is not merely a prodrome to vomiting but a distinct subjective experience capable of inducing aversion and avoidance behaviors via independent ascending pathways. Recent work by Zhang et al. (2021) utilizing single-cell transcriptomic analysis has demonstrated this clinical distinction at the molecular level. They identified genetically distinct cell populations (e.g., GFRAL/GLP1R-expressing neurons) within the area postrema, adjacent to the nucleus tractus solitarii, that are dedicated to mediating nausea and food avoidance behaviors. This confirms that the circuitry for nausea and vomiting is anatomically and molecularly segregated from the simple pharyngeal defensive reflex circuitry of the gag.

Finally, dysgeusia is associated with integrative dysfunction in the rostral nucleus tractus solitarii. Distortion of taste perception persisting long after damage to peripheral taste receptors (e.g., following viral infection or chemotherapy) reflects not simply the absence of receptors, but a maladaptive reconfiguration within high-order gustatory networks connecting the rostral nucleus tractus solitarii with the thalamus and insular cortex (Moe et al., 2024). Such circuit distortion often leads to poor appetite, food avoidance, and severe nutritional imbalance.

In summary, clinical symptoms associated with the nucleus tractus solitarii cannot be understood solely as the result of damage to a single nucleus. They represent a hierarchical process in which alteration of peripheral sensation disrupts the integrative principle within the nucleus tractus solitarii, which in turn leads to failure of supranuclear modulation, ultimately becoming fixed as abnormal behavioral manifestations (hypersensitivity or unresponsiveness) (Benarroch, 1993; Hamdy et al., 1998; Humbert and German, 2013).

## Neuromodulation and future directions: circuit reconfiguration via patterned input

The recognition that the nucleus tractus solitarii (NTS) serves as a crossroads for sensory convergence, integration, and supranuclear modulation suggests that this region represents a multimodal therapeutic target for neurological and internal medical disorders. Current therapeutic strategies are evolving beyond simple symptomatic relief toward integrated approaches that combine electrical stimulation (e.g., VNS), molecular targeted therapy, and sensorimotor rehabilitation to reconfigure NTS-centered pathophysiological circuits (Table 6).

**TABLE 6:** Neuromodulatory approaches and future directions targeting NTS circuits.

Modality	Target and mechanism	Potential clinical application
Vagus nerve stimulation (VNS)	Patterning of afferent input → opening of plasticity gate; activation of anti-inflammatory pathway	Stroke rehabilitation, rheumatoid arthritis, epilepsy
Pharmacological modulation	Targeting P2X3 (cough) and GLP-1 (metabolism) receptors	Refractory chronic cough, obesity, and metabolic syndrome
Sensory-based rehabilitation	Peripheral (pharyngeal/laryngeal) sensory stimulation (PES, thermal)	Induction of swallowing reflex recovery in dysphagia patients
Cortical neuromodulation	rTMS/tDCS → enhancement of descending control	Restoration of volitional control over swallowing/coughing
Future: closed-loop systems	Stimulation based on real-time vagal response monitoring	Precision neuromodulation tailored to individual states

This table summarizes therapeutic strategies utilizing the NTS as a hub for plasticity and anti-inflammation (Dawson et al., 2021; Engineer et al., 2011; Winston et al., 2021; Genovese et al., 2020; Hulsey et al., 2017; Pavlov and Tracey, 2012; Roosevelt et al., 2006; Smith et al., 2020; Tracey, 2002).

Traditionally, the anticonvulsant effects of vagus nerve stimulation (VNS) were explained by a mechanism involving the modulation of whole-brain neurotransmitter levels via projections from the NTS to the locus coeruleus (LC) and dorsal raphe nucleus (Groves and Brown, 2005; Roosevelt et al., 2006). However, recent work by Engineer et al. (2011) and Hulsey et al. (2017) demonstrated that VNS recruits the NTS–LC–basal forebrain axis to open a plasticity gate in the cortex (Engineer et al., 2011; Hulsey et al., 2017). This implies that when VNS is precisely paired with motor rehabilitation training following stroke, it can dramatically accelerate the functional recovery of damaged cortical regions (Dawson et al., 2021).

Furthermore, the NTS holds significant therapeutic implications as the starting point of the cholinergic anti-inflammatory pathway, central to brain–immune interactions. In particular, the anatomical fact that approximately 80% of vagus nerve fibers are afferent and terminate in the NTS suggests that the therapeutic mechanism of VNS should be understood not merely as direct electrical activation, but as the delivery of patterned afferent input that remodels NTS network activity. This concept was recently supported by the landmark clinical study of Genovese et al. (2020). They demonstrated significant anti-inflammatory effects by applying a miniaturized VNS device to patients with multidrug-refractory rheumatoid arthritis, marking a significant advance showing that neuromodulation can be established as a substantive disease-modifying anti-inflammatory therapy rather than simple symptom alleviation (Genovese et al., 2020; Pavlov and Tracey, 2012; Tracey, 2002). Such approaches are likely to extend to other refractory inflammatory conditions, including inflammatory bowel disease (IBD), in the future.

In parallel, molecular targeted therapies aimed at specific receptors within the NTS are being actively researched. Notably, gefapixant, a P2X3 receptor antagonist, effectively controls refractory chronic cough by inhibiting the hypersensitivity of vagal afferent fibers, thereby restoring cough gating at the level of the NTS (Smith et al., 2020). Additionally, the aforementioned GLP-1 receptor agonists directly stimulate the satiety signaling system of the NTS, offering a dual action of treating obesity and inducing central anti-inflammatory effects (Holt, 2022). These pharmacological approaches can generate synergy when combined with behavioral interventions that strengthen peripheral sensory input to induce NTS plasticity without invasive stimulation. For instance, pharyngeal electrical stimulation (PES) or thermal–tactile stimulation used in dysphagia treatment activates oropharyngeal sensory receptors to increase the excitability of the swallowing CPG within the NTS and facilitate the reorganization of the swallowing center in the cortex (Hamdy et al., 1998).

Future research is expected to develop closed-loop systems that optimize electrical, pharmacological, and behavioral interventions by monitoring individual NTS circuit sensitivity (e.g., vagal evoked potentials) in real time to realize precision neuromodulation (Winston et al., 2021). An integrated understanding of the NTS, ranging from developmental origins to clinical pathophysiology, will be key to proposing new therapeutic solutions for refractory nervous system disorders.

## Conclusion

The nucleus tractus solitarii (NTS) acts as a central platform for visceral sensory integration conserved across vertebrates, maintaining a fundamental integrative structure that transforms sensory inputs into autonomic and motor outputs. The functional expansion observed in humans can be understood as the result of a supranuclear regulatory layer superimposed upon conserved brainstem circuits, rather than the emergence of new circuitry. Clinically, symptoms such as dysphagia, cough hypersensitivity, nausea/vomiting, and dysgeusia need to be reinterpreted not as failures of single reflexes, but as regulatory imbalances of the sensory–motor–autonomic network centered on the NTS. This perspective extends the understanding of neuromodulation strategies from simple electrical stimulation to the regulation of patterned afferent inputs that induce plasticity in NTS circuits. Such a multi-layered approach will provide a foundation for a more systematic understanding of the pathophysiology of brainstem-centered diseases.
